# Supplementary material for: Effects of Three Different Doses of Inter-Alpha Inhibitor Proteins on Severe Hypoxia–Ischemia-Related Brain Injury in Neonatal Rats
Source: Int J Mol Sci. 2022 Nov 3;23(21):13473. doi: 10.3390/ijms232113473 (PMC9655902; doi:10.3390/ijms232113473)
Supplement: Supplementary file 1 [file ijms-23-13473-s001.zip › ijms-1885904-supplementary.pdf]

Table S1. Behavioural analyses in neonatal rats after exposure to hypoxia-ischemia and treatment with human inter-alpha inhibitor proteins (hIAIPs).

Behavioural Test	Unit	Male					Female				
		Sham	HI-PL	HI-30	HI-60	HI-90	Sham	HI-PL	HI-30	HI-60	HI-90
Righting Reflex	Seconds	3 ± 1	6 ± 3	3 ± 2	4 ± 1	3 ± 2	3 ± 2	5 ± 3	4 ± 3	4 ± 2	4 ± 2
Negative Geotaxis – time to turn	Seconds	9 ± 7	6 ± 3	8 ± 10	6 ± 2	6 ±4	9 ± 4	7 ±5	7 ± 3	8 ± 4	6 ± 5
Open Field – Zones Entered	%	97 ± 10	85 ± 17	91 ± 21	89 ± 18	79 ± 21	91 ± 21	66 ± 21	79 ± 16	94 ± 11	84 ± 20
Open Field – Postural changes	Occurrences	250 ± 132	454 ± 173	319 ± 153	277 ± 151	226 ± 122	259 ± 118	221 ± 153	189 ± 146	383 ± 241	294 ± 231
Open Field – Distance Travelled	cm	1544 ± 365	1595 ± 281	1840 ± 375	1682 ± 408	1599 ± 416	1396 ± 445	1323 ± 395	1348 ± 356	1725 ± 412	1664 ± 422
Open Field – Maximum acceleration	cm/s^2	2259 ± 865	2166 ± 922	2664 ± 1409	3711 ± 5923	2575 ± 1270	2371 ± 918	1872 ± 864	1602 ± 663	2832 ± 1378	2240 ± 1079
Open Field – Average Velocity	cm/s	3 ± 1	3 ± 1	3 ± 1	3 ± 1	3 ± 1	2 ± 1	2 ± 1	2 ± 1	3 ± 1	3 ± 1
Open Field – rotation clockwise	Occurrences	7 ± 3	11 ± 4	12 ± 6	9 ± 5	11 ± 6	7 ± 3	10 ± 6	7 ± 3	14 ± 6	11 ± 4
Open Field – rotation anti-clockwise	Occurrences	7 ± 3	7 ± 4	7 ± 3	8 ± 3	10 ± 4	7 ± 3	6 ± 4	7 ± 2	9 ± 5	11 ± 5
Open Field – Time in outer 12 segments	Seconds	505 ± 79	480 ± 66	504 ± 43	462 ± 111	514 ± 41	532 ± 38	459 ± 172	521 ± 38	455 ± 152	396 ± 189
Open Field – Time in centre four segments	Seconds	87 ± 76	104 ± 58	82 ± 40	133 ± 111	81 ± 42	55 ± 37	134 ± 176	71 ± 30	141 ± 154	174 ± 189

Sham placebo treated (Sham)
Hypoxic-ischemic placebo treated (HI-PL)
Hypoxic-ischemic 30 mg/kg IAIP treated (HI-30)
Hypoxic-ischemic 60 mg/kg IAIP treated (HI-60)
Hypoxic-ischemic 90 mg/kg IAIP treated (HI-90)
Values are mean ± SD
